# SEM Analysis and Management of Fracture Dental Implant

**DOI:** 10.1155/2013/270385

**Published:** 2013-04-23

**Authors:** Archana Singh, Ankita Singh, Rajul Vivek, T. P. Chaturvedi, Pankaj Chauhan, Shruti Gupta

**Affiliations:** Department of Prosthodontics, Faculty of Dental Sciences, Institute of Medical Sciences, Banaras Hindu University, Varanasi 221005, India

## Abstract

Implant fracture is one of the important biomechanical complications which can present with a considerable problem to the patient as well as the dental surgeon. The aim of this case report is to describe the management of a case of fractured endosseous dental implant in premolar region and microscopic evaluation of the fractured implant segment using scanning electron microscopy. In most of such cases, complete removal of the fractured implant has been a preferred treatment option. In the present case, fractured implant segment was successfully removed and rehabilitated immediately with larger diameter implant. It was found that retrieved fracture segment had a diameter of 3.3 mm, and SEM analysis shows fatigue fractures which may be the result of excessive overloading and use of small diameter implant which enhances fatigue failure.

## 1. Introduction

Dental implants have been a preferred treatment option for rehabilitation of completely and partially edentulous patients. A major concern for dentist and patient is the durability of the dental implantation. Although the success rate of this treatment is more than 90% [1], the incidence of implant fracture has been reported in 0.16–1.5 percent of the cases [2]. 

One of the major causes of implant fracture is biomechanical overloading which occur due to various parafunctional activities like bruxism, inadequate occlusion, the presence of distal extensions or cantilevers, and lack of prosthetic passive fit over the implants resulting in metal fatigue [3–7]. Other causes may be peri-implant vertical bone loss [8, 9] due to peri-implantitis and occlusal trauma; galvanic corrosion may be an additional causative factor contributing to implant fracture [10]. Management of a case of implant fracture may pose a challenge to the clinician because of its surgical, rehabilitative, and emotional implications.

The aim of this case report is to describe the management of a case of fractured endosseous dental implant and microscopic evaluation of the fractured implant segment using scanning electron microscopy (SEM).

## 2. Case Report

**Figure 1:**
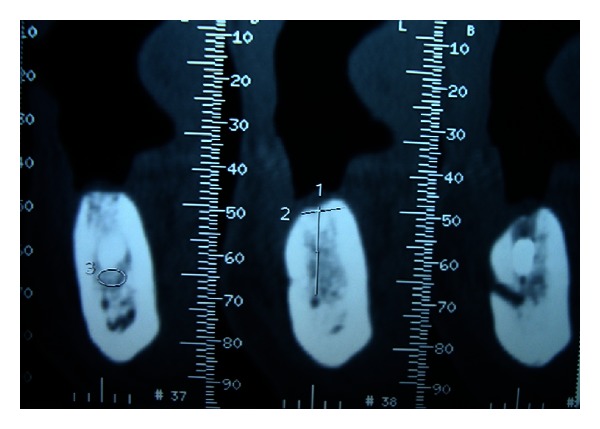
Preoperative Dentascan.

A 45-year-old female patient reported to the department with loss of coronal part of implant-supported prosthesis in the lower left premolar region. Detailed history revealed successful implant placement 3 years back in mandibular left second premolar region. Medical history of the patient was found to be noncontributory. Development of pain and mobility of the prosthesis started since the last few months with loss of mobile segment one week back. On radiographic examination, Dentascan revealed remaining part of the implant fixture to be still embedded in the bone (Figure 1).

**Figure 2:**
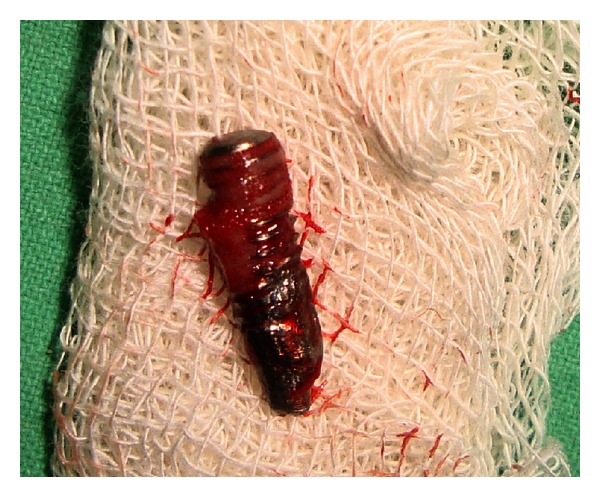
Surgically retrieved implant fragment.

**Figure 3:**
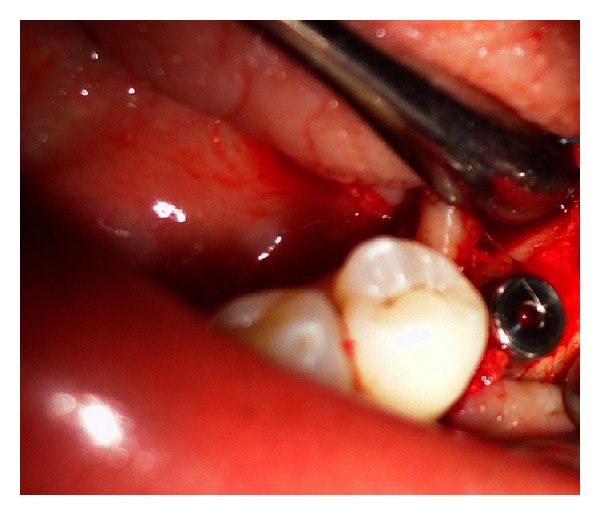
Implant placed at mandibular left second bicuspid region.

Treatment plan for the removal of remaining implant fixture, the placement of a new implant of a larger diameter immediately, and consecutive prosthetic rehabilitation was discussed. After obtaining patient consent, the surgical phase was initiated. Under local anesthesia, a mucoperiosteal flap was elevated exposing the fractured implant which was removed by a trephine bur (Figure 2). This was followed by preparing the osteotomy site for placement of a new implant of larger diameter of 5 × 11.5 mm (Hi-tech tapered, self-threaded, Life Care Device private limited, Israel). The recommended drills were used in required sequence, the endosseous implant was placed immediately after removing the fracture segment (Figure 3) with initial stability of 40 N, and mucoperiosteal flap was approximated with interrupted sutures. The patient was put on antibiotics, anti-inflammatory analgesics, and oral rinses for a week. 

**Figure 4:**
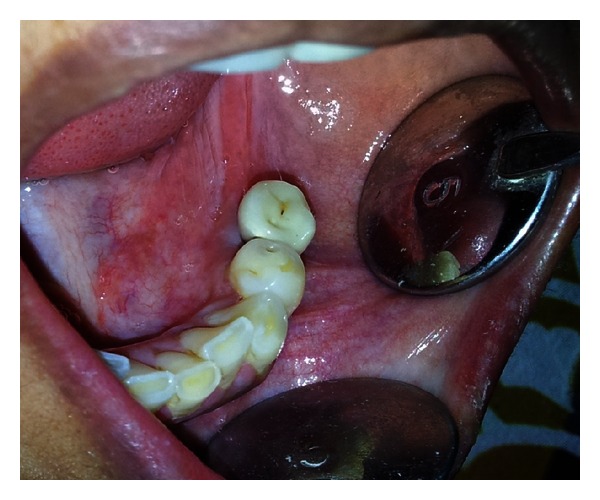
Fixed ceramometal prosthesis is placed (occlusal view).

**Figure 5:**
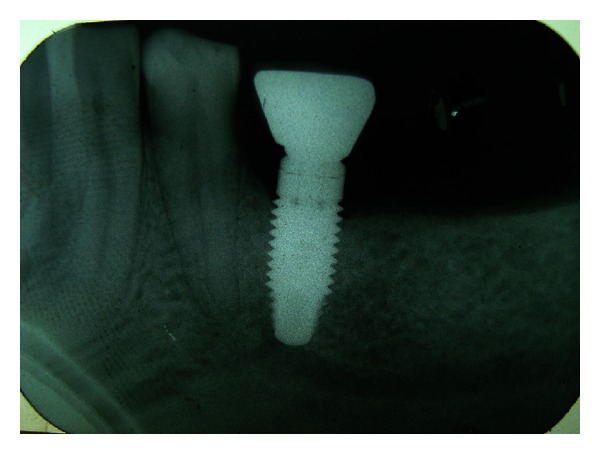
IOPA radiograph 3 months after prosthetic rehabilitation.

**Figure 6:**
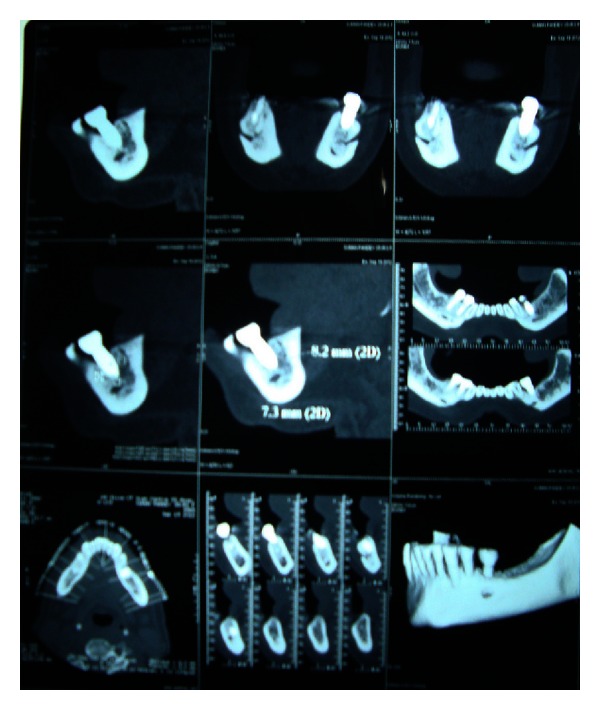
Dentascan 12 months after prosthetic rehabilitation.

Second stage surgery was done after 4 months. The prosthetic phase was initiated by impression making use of open window technique with polyvinyl silicone impression material (Aquasil Dentsply/Caulk, Milford, DE, USA). Abutment preparation and try-in were done, and working cast was sent to the laboratory for fabrication of ceramometal prosthesis (Dentsply, Ceramco, York, PA, USA). Consecutively, the implant was loaded with cement-retained metal-ceramic crown (Figure 4). Reports of 36-month followup at every 3, 6, and 12 months interval have shown successful results so far (Figures 5 and 6).

**Figure 7:**
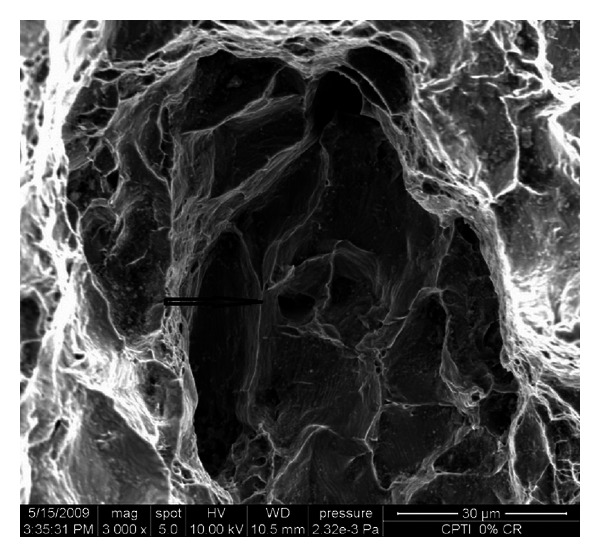
Scanning electron micrograph shows intergranular fracture of implant (large dimple at the center of implant surface is shown by arrow).

The retrieved part of implant fixture (Figure 2) was sent for SEM analysis (Figure 7) for the probable cause of the fracture. Scanning electron micrograph of fracture implant surface shows an extent evidence of intergranular fracture. A large dimple at the centre of the implant surface was found which consists of various wavy lines or striations.

## 3. Discussion

Success and survival rates of osseointegrated dental implant have been reported close to 90–95% [1]. Although the success rate is high, one of the infrequent yet important causes of failure of dental implant is fracture. The incidences of implant fracture reported by Pylant et al. and Goodacre et al. are 0.98%, and 1.5%, respectively [4, 11]. 

A number of factors should be considered while analyzing the reasons of fractured dental implants. This may include an excessive occlusal load, location of the implant, an insufficient number of implants supporting the prosthesis, the material from which the prosthetic screws are made, and an implant diameter of under 3.75 mm [12].

Rangert et al. [3] reported that 90% of fractured implants are located in the region of molars and premolars. Balshi [13] found that all implant fractures occur in the area of premolars and molars, and no distinction has been made between the upper and lower jaws. Gargallo et al. [14] reported similar results with 80.9% fractured implants located in the molar and premolar region within 3-4 years after loading. This result is in agreement with our case where implant fracture was reported 3 years after implant placement in the mandibular second premolar region. In the present case report, the diameter of the retrieved implant was found to be 3.3 mm. Small diameter of implant <3.75 mm may be another factor contributing to the failure as reported by various studies [13, 15, 16]. According to Shemtov-Yona et al. 3.3 mm diameter implants did not exhibit a typical fatigue behavior like 5 and 3.75 mm implants; that is, implants were fractured at the abutment neck and screw region. In 3.3 mm implants, 52% of the fractured implants were fractured at the implants second thread and 48% were fracture at the implants third thread. This result is in agreement with our case [17]. 

Three options for management of implant fracture have been reported in literature [4, 13, 18].

1. Complete removal of the fractured implant using explanation trephines.
2. Removal of the coronal portion of fractured implant with the purpose of placing a new prosthetic post.
3. Removal of the coronal portion of the fractured implant, leaving the remaining apical part integrated in bone.

Complete removal of fractured implant was the preferred treatment option for this patient. In this case complete removal the fractured implant was done, placing a newer larger diameter implant immediately in same surgical bed. External diameter of the trephine bur used for the removal of fractured implant was also kept in mind while selecting the diameter of new implant to ensure primary stability. The retrieved fractured implant was further sent for SEM analysis to study the microscopic features and to investigate the cause of implant fracture.

## 4. SEM Analysis of Fractured Implant Fragment

SEM image of fractured implant surface was shown in Figure 7. Although the fractured surface is rather complex, the surface shows to an extent evidence of intergranular fracture. A large dimple at the centre of the implant surface was found which consists of various wavy lines. These lines are considered to be the slip bands formed by repeated loading in the mouth. Excessive overloading produces increase in number of dislocation, which, by virtue of their interactions and stress field, gives rise to higher state of internal stresses and consequently leads to fatigue fracture. Shemtov-Yona et al. [19] also proposed that the nontypical fatigue behavior observed for 3.3 mm implant diameter is probably the result of stress concentrations generated along the structure's surface.

## 5. Conclusion

Dental implant fracture is an infrequent yet important cause of implant therapy failure, and adequate measures should be adopted to prevent it. In this case, it was concluded that cause of implant fracture was found to be metal fatigue due to repeated overloading and use of small diameter implant.
